# A Signature of Attractor Dynamics in the CA3 Region of the Hippocampus

**DOI:** 10.1371/journal.pcbi.1003641

**Published:** 2014-05-22

**Authors:** César Rennó-Costa, John E. Lisman, Paul F. M. J. Verschure

**Affiliations:** 1 Universitat Pompeu Fabra, Synthetic, Perceptive, Emotive and Cognitive Systems group (SPECS), Barcelona, Spain; 2 Federal University of Rio Grande do Norte (UFRN), Brain Institute (ICe), Natal, Rio Grande do Norte, Brazil; 3 Brandeis University, Biology Department & Volen Center for Complex Systems, Waltham, Massachusetts, United States of America; 4 Catalan Institute of Advanced Research (ICREA), Passeig Lluís Companys 23, Barcelona, Spain; 5 Universitat Pompeu Fabra, Center of Autonomous Systems and Neurorobotics (NRAS), Barcelona, Spain; Indiana University, United States of America

## Abstract

The notion of attractor networks is the leading hypothesis for how associative memories are stored and recalled. A defining anatomical feature of such networks is excitatory recurrent connections. These “attract” the firing pattern of the network to a stored pattern, even when the external input is incomplete (pattern completion). The CA3 region of the hippocampus has been postulated to be such an attractor network; however, the experimental evidence has been ambiguous, leading to the suggestion that CA3 is not an attractor network. In order to resolve this controversy and to better understand how CA3 functions, we simulated CA3 and its input structures. In our simulation, we could reproduce critical experimental results and establish the criteria for identifying attractor properties. Notably, under conditions in which there is continuous input, the output should be “attracted” to a stored pattern. However, contrary to previous expectations, as a pattern is gradually “morphed” from one stored pattern to another, a sharp transition between output patterns is not expected. The observed firing patterns of CA3 meet these criteria and can be quantitatively accounted for by our model. Notably, as morphing proceeds, the activity pattern in the dentate gyrus changes; in contrast, the activity pattern in the downstream CA3 network is attracted to a stored pattern and thus undergoes little change. We furthermore show that other aspects of the observed firing patterns can be explained by learning that occurs during behavioral testing. The CA3 thus displays both the learning and recall signatures of an attractor network. These observations, taken together with existing anatomical and behavioral evidence, make the strong case that CA3 constructs associative memories based on attractor dynamics.

## Author Summary

A type of neural network called an “attractor network” is thought to underlie memory associations. Importantly, when such a network is presented with part of a memory, the network activity is attracted to the complete memory. However, it has been difficult to obtain clear experimental evidence for such attractor networks. Indeed, recent “morphing” experiments that were specifically designed to observe these attractor dynamics in the hippocampus did not obtain the expected results, leading to a controversy on the validity of the attractor hypothesis of memory. Here, we have built a computational model of the relevant hippocampal areas, including its core anatomical and physiological features, and through the use of large-scale computer simulations reveal in detail the physiological properties expected of the hippocampal attractor network during morphing experiments. We show that the experimental results obtained are actually those to be expected of an attractor network when the specifics of the experimental protocol are taken into account. Most importantly, the results directly demonstrate the attraction of CA3 activity to a stored pattern. Our results, together with previous behavioral and *in vitro* studies, provide strong evidence that CA3 is an attractor network for associative memory.

## Introduction

Theoretical work has shown how networks with excitatory recurrent connections can function as an associative memory [1]–[4]. Specifically, Hebbian plasticity at the synapses of recurrent connections leads to the association of the elements of a memory. Information stored in this way can be recalled given external input of a partial pattern, thus displaying “pattern completion” [5], [6]. This “attraction” of network activity to a stored pattern provides a useful form of associative memory and has inspired much theoretical and experimental work. Among hippocampal sub-regions, CA3 is unique in having extensive excitatory recurrent connections [7], [8]. This property, together with the finding that the synapses of these recurrent connections can undergo Hebbian plasticity [9], [10], has led to the hypothesis that CA3 has attractor dynamics and serves as the main site for associative memory storage in the hippocampus [11]–[16].

Despite the influence of the attractor concept, it has been difficult to obtain direct experimental support for attractor networks in the hippocampus. Experiments specifically designed to observe the electrophysiological signature of attractor dynamics in CA3 have been problematic (for a review, see [17]). The experiments designed to identify attractors first established memories of two environments with different shapes (square/round) but unaltered distal cues; the environment was then gradually morphed from one to the other [18]–[20]. Given that attractor networks can display winner-take-all dynamics, the expectation was that, during such morphing, the network would first be attracted to one stored memory and would then make a sudden transition to the other. Because the firing patterns in CA3 changed gradually rather than suddenly, it has been argued that the results are inconsistent with the properties of attractor dynamics and that a different function of CA3 should be entertained [21]. Alternatively, it has been suggested that sudden transitions may not be an appropriate criteria for identifying attractor networks [22]. Here, we directly address this issue, which is central to understanding the hippocampal contribution to associative memory.

We have developed a computational model of CA3 and its input structures and have used this model to simulate the morphing experiments described above [18]–[20]. This model was constrained not only by the properties of CA3, but also by the properties of the input to CA3 from the dentate gyrus (DG) and entorhinal cortex (EC). Furthermore, because CA3 cells fire selectively during only a small fraction of the theta cycle [23], [24], we modeled the dynamics of CA3 that could occur in a comparable short time segment. With this model, we have been able to account for the results obtained during morphing experiments and to analyze how the CA3 recurrent connections affect network function and dynamics. Our analysis clarifies the criteria that should be applied to identify attractor dynamics under the conditions of morphing experiments. These criteria are satisfied by the data. Our simulations show that CA3 only satisfies these criteria when recurrent excitation is present, leading us to conclude that intra-CA3 processes in fact support attractor dynamics. Notably, for small morphs, the pattern of activity in CA3 is attracted to a stored pattern, whereas the pattern in DG, a region that provides input to CA3, is not. We have also analyzed an additional experimental observation, the hysteresis observed in CA3 recordings during morphing [19]. Our analysis suggests that this hysteresis arises from CA3 plasticity, thus suggesting a new method for observing how experience affects CA3 attractor dynamics.

## Materials and Methods

### EC/DG/CA3 network model

**Figure 1:**
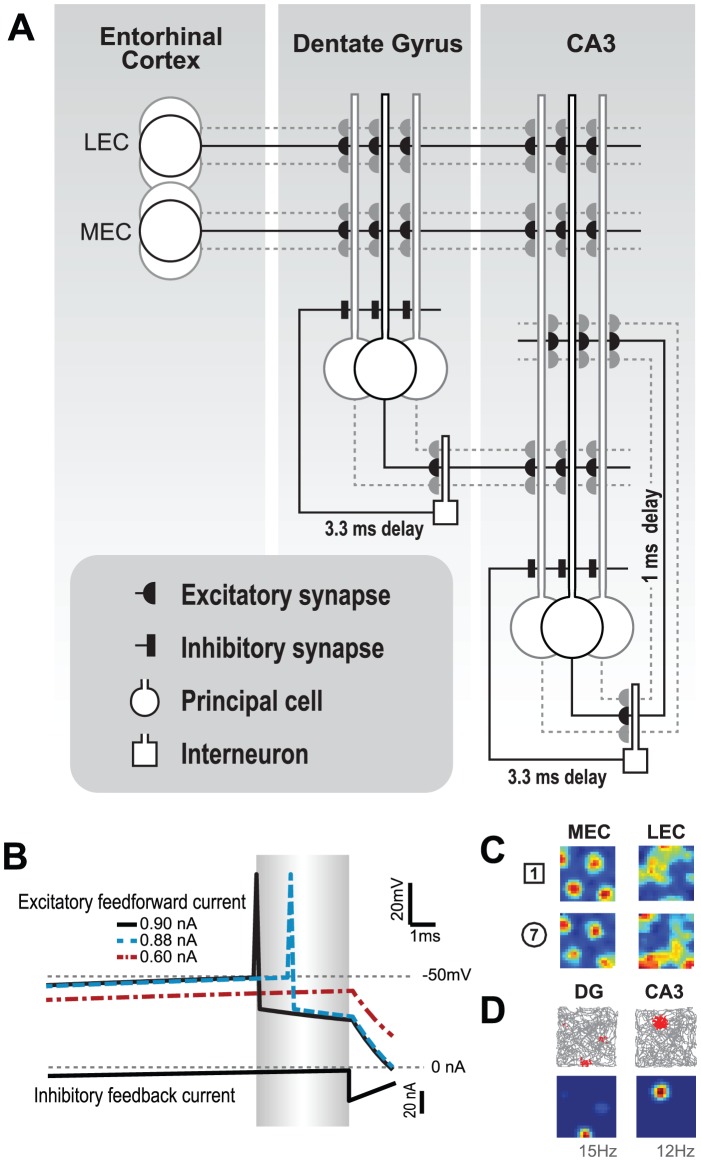
The EC/DG/CA3 model. (**A**) In our model of the EC/DG/CA3 system, the excitatory granule cells of DG receive convergent input from EC (2700∶1) combined with a delayed feedback inhibition (delay: 3.3±0.4 msec) imposed by local interneurons. Excitatory cells in CA3 receive convergent input from both EC (2900∶1) and DG (∼50∶1) together with delayed feedback inhibition from local interneurons (delay: 3.3±0.4 msec) and recurrent excitatory input (delay: 1 msec). (**B**) Delayed feedback inhibition mediates internal competition that selects which cell fires in a given gamma cycle. Trace of three sample cells with different strength of excitatory feedforward currents. Time is represented at the horizontal axis. Gray area designates the window between the first spike and the onset of global inhibition. Cell voltage and input currents are shown on the ordinate. (**C**) Rate maps of sample EC neurons shown for both extreme shapes. (**D**) Action potentials (red dots) with overlaid trajectory (gray line) and equivalent rate maps of sample DG and CA3 cells.

The model that we developed is illustrated in Figure 1A. We modeled pyramidal cells of CA3 (N_CA3_ = 10,000) and the granule cells of the dentate gyrus (DG, N_DG_ = 800,000) as one-compartment integrate-and-fire neurons [25]. The voltage 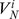 of each neuron *i* is determined by the input feedforward excitatory input, I_FF_, the recurrent excitatory input, I_REC_, the recurrent inhibitory input, I_GABA_, and the after-hyperpolarization, I_AHP_, currents. Both DG and CA3 cells receive feedforward excitatory current from the EC (N_EC_ = 160,000), I_EC_, and recurrent inhibition from their respective interneuron networks, I_GABA_. CA3 cells also receive feedforward excitatory current from DG cells, I_DG_, and recurrent excitatory current from the recurrent collaterals of CA3, I_CA3_. We use as parameters the average input resistance (R_n_ = 33 MΩ) [26], the membrane time constant (τ_n_ = 30 ms), and the firing threshold (T = −50 mV). Voltage is reset to rest (V_REST_ = −65 mV) after each spike. The after-hyperpolarization maximum current is set to A_AHP_ = −2 nA with τ_AHP_ = 7 ms decay [27]. To emulate an absolute refractory period caused by sodium channel inactivation, cells that emitted a spike were not allowed to spike for the following period τ_SPIKE_ = 2 ms. Inhibition is global within each region (CA3 and DG) and occurs with a delay of 3.3±0.4 msec [28] relative to the first spike succeeding the previous inhibitory current discharge [29]. The membrane potential of neuron *i* evolves according to:
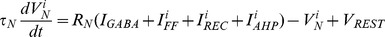
(1.1)


The feedforward excitatory input current of cell *i*, 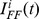, is computed through the arithmetic sum of all excitatory post-synaptic currents from synapses reaching *i* multiplied by a feedforward gain factor 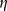. For DG cells: 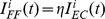; while for CA3 cells: 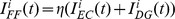. 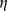 was estimated as 0.68 nA in order to allow network oscillation within gamma frequency (∼35 Hz).

The entorhinal input current, I_EC_, of each CA3 and DG cell is computed as a linear combination of the inputs from the lateral and medial entorhinal cortices regulated by a mixing factor α (Equation 1.2). There is no data available to directly estimate α, so we quantitatively estimate it through a parametric search methodology. The inputs of the lateral and medial entorhinal cortices are computed independently and are normalized by the mean maximum value considering all positions (Equations 1.3 and 1.4). Normalization of the input allows an interpretation of α with respect to the overall size of the EPSC originated in each of the entorhinal cortices. Feedforward synaptic weights, W_MEC_ and W_LEC_, are randomly assigned from a distribution that corresponds to the measured distribution of synapse size [30], [31]. The number of EC cells that converge into the DG and CA3 can be estimated from the measured spine density of 2.3 spines/µm and dendrite length of 3000 µm [32]. Considering that each spine has one synapse, we estimate for the measured spine density and dendritic length [33] that each DG cell receives input from 1200 MEC and 1500 LEC cells, while CA3 cells receive inputs from 1400 MEC and 1500 LEC cells [34], [35]. To emulate the morphing experiment (see below), the total EC input of each cell *i*, *I^i^_EC_*, is defined for each of the *N_r_* positions *r(x,y)* and *N_c_* wall shapes *c*:
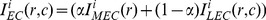
(1.2)

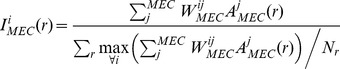
(1.3)

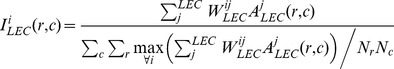
(1.4)


The DG input current to CA3 cells is set respective to the normalized activity of a randomly selected presynaptic DG cell multiplied by a relative gain factor β. Activity of DG cells is computed *a priori* as a mean rate (see Data analysis section) and is set constant for a specific position and morphing stage. The input of the DG to CA3 neuron *i* is defined as:
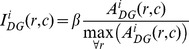
(1.5)


The recurrent CA3 input current of cell *i*, 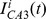 is determined by the non-linear threshold function of the arithmetic sum of the recurrent excitatory current, 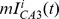: 
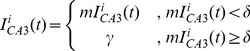
(1.6)where 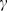 = 20 nA is the asymptotic feedback excitatory current and 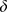 is the recurrent excitation threshold, considering a non-linear mapping from input to excitatory current [36]. Recall threshold is defined as the threshold complementary 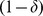. 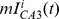 is computed as the sum of the product of the afferent activity, 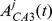, and the specific synaptic weight, 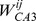. 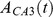 is modeled as a step function with 3 msec duration and release lag, 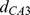, of 1 msec [37]: 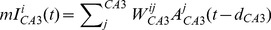
(1.7)


### Environment morphing experiment

To emulate the experimental procedure and the ensuing hippocampal neuronal dynamics, the activity of the model was computed over the trajectory of real rats available online [38]. All trajectories were obtained from sessions in a square environment, and morphing was encoded in the activity of LEC neurons. We do not implement any direct source of noise in our simulations. Indirectly, noise arises naturally as fluctuation of EPSPs from the combination of a high-resolution spatial representation (computed bins of 1 cm^2^ compared to place fields of >200 cm^2^) with the natural tracking of the position of the animal. Noise also arises from the IPSP delay, which is set probabilistically with a normal distribution. Neural activity was computed over sessions of 10 minutes (T = 600 sec). Each gamma cycle (t = 36.5 msec) was computed independently with a randomly selected IPSP delay determined by a normal distribution with specific SD (d = 3.3±0.4 msec) and with the potential of all neurons initialized at rest (Figure 1B). Different cells could assume multiple rate values (see Data analysis section) because the release of action potentials was probabilistic: the cells with the strongest input fire in every such simulated cycle and thus show a high probability (rate, λ). Cells that fire just after the strongest cell will fire in most simulated cycles approximating the maximum rate, whereas cells with less excitation may fire late in the gamma cycle but often do not fire at all, thus displaying a low rate. Recurrent excitation is applied within the active window of the gamma cycle and can cause a cell with low feedforward excitation to produce an action potential, increasing its rate.

Activity of EC cells is dependent on the current position of a virtual rat, *r(x,y)*, which navigates through the environment following empirically determined trajectories of real rats (see below), and the current progression in the morphing procedure, *c*. The computation is analogous to previous work [34]. The activity of MEC cells is defined by a mathematical description of grid cells [39] and is made insensitive to morphing [19], [40] (Figure 1C, left), unless when noticed otherwise. To simulate the conditions that lead to global remapping in the CA3 [21], [40], grid cell realignment is implemented by setting a different angular and position phase but letting the same spatial frequency. Both MEC and LEC rate maps are tailored to fit the observed spatial information score [41]. Morphing is encoded in the activity of the LEC cells by allowing a sharp transition between two independent rate maps at a morphing degree specific for each cell defined randomly following a uniform distribution [34] (Figure 1C, right). The LEC selectivity to morphing is grounded in the observed selectivity of LEC cells to objects [42], [43], the fact that it receives strong input from sensory driven areas [44]–[46], and the finding that rate remapping in the CA3 is impaired by LEC lesions [20]. Importantly, although we assume a sharp transition for the response of LEC cells during morphing, the fact that the point of the transition is different for each cell and that the activity of many cells is summed makes our implementation equivalent to the case in which each cell had a smooth or non-uniform response to morphing. LEC and MEC activity was produced with a resolution of 1 cm×1 cm.

Excitatory input for the EC and DG was computed using the virtual rat's position as a reference (x,y). The model was updated with a step size of 1 msec from the beginning of the gamma cycle and the time of the first spike and 0.1 msec steps in the interval between the first spike and the release of GABA. Spikes were time stamped for further analysis. Due to the expected low activity levels of the DG [47], [48], only the cells with mean weight strengths within the upper 10% percentile were simulated.

To emulate the rat's exposure to the square and round environments, the recurrent CA3 weight matrix is defined based on the history of the firing of cells on the square and round environments without recurrent excitation. As we are not interested in the dynamics of plasticity prior to morphing experiment, we used an interleaved procedure to define the recurrent CA3 weight matrix as follows: to enhance orthogonalization of CA3 activity following the CA3-DG interaction [16], each CA3 cell is assigned to a cluster through a k-means algorithm using spatial correlation as a distance metric. The number of clusters is set to maximize the grouping of the data [49]. Cells belonging to the same cluster (C) are interconnected with a weight inversely proportional to the size of the cluster (n(C)) so that the sum of all synaptic weights to each cell is equal to 1:
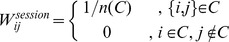
(1.8)


The training is performed for the two extreme shapes of the environment, and the synaptic weight between two cells is defined as the maximum value over the two conditions: 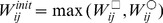
(1.9)


Recurrent CA3 weights are updated at the end of every session: a temporary connection matrix is built using the above clustering method, and the weights interconnecting active cells are updated by a convex sum-ruled by a learning factor (L_RATE_) between the previous weights and the weights in the temporary connection matrix:
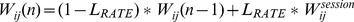
(1.10)


### Data analysis

Data analysis includes construction of 16×16 bin rate maps, place fields analysis, population vector correlation, rate overlap, and spatial correlation and is performed following the same procedures and methods as reported for the experimental data [19]. In summary, the outcome of the simulations was a list of time-stamped spikes that could be related to the r(x,y) coordinate in which they were emitted (r(t)). Space was discretized in 16×16 bins (N_r_ = 256, equivalent to 5×5 cm). For the specific case of the DG input to CA3 and the activity map of MEC and LEC cells, space was discretized in 80×80 bins (N_r_ = 6400, equivalent to 1×1 cm). For each bin, the firing rate was calculated by averaging the number of spikes at a certain position and dividing it by the average occupancy of that bin (Figure 1D). Rate maps were smoothed by a Gaussian kernel (g) of h = 5 cm sd: 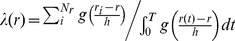
(1.11)


Cells with a mean firing rate above 0.1 Hz in at least one of the morphing steps were considered active. Place fields were determined by the existence of continuous bins (n>8 and n<128) with a peak rate no less than 2 Hz, with all units above 20% of this peak value.

The population vector (PV) correlation was calculated by correlating the response vector of all cells in a specific bin and correlating it to: (a) the same response vector under a different morphing condition and (b) a response vector of a different bin localized 50 cm away under the same morphing condition. Only active cells with a firing rate above 1 Hz in the two conditions were considered for the PV.

The overlap between two rate maps was measured by dividing the mean rate displayed in the less active condition by the mean rate in the more active condition. The spatial correlation was defined as the pair-wise correlation of the rate maps considering each bin. In our simulations, to correct for sampling error, all comparisons in the morphing experiment between rate maps were performed using simulation data from different trajectories.

## Results

In the experiments of Leutgeb et al. [18], [19], the walls defining the environment were morphed from a square shape (1) into a circular shape (7) over five intermediate shapes (2–6) while distal cues were kept constant. Morphing occurred after the animals were initially exposed to both the square and circular environments, thereby establishing a memory for these extremes. The classic notion of attractor dynamics specifies that there will be an abrupt transition in the cell firing pattern as the environment is gradually morphed [21]. However, no such transition was observed (Figure 3A of [19]). To the contrary, the population vector (PV) correlation, which can be used to quantify the difference in recorded CA3 population responses in two different environments, changed gradually. These changes were caused by alterations in the cells' peak firing rate (either up or down) without modification of the identity of the active cells, a process called rate remapping [19], [34]. Importantly, DG and CA3 behaved differently; for the smallest morphs (environments 1 to 2 or 7 to 6), the change in the PV correlation was much larger in the DG than in CA3 (Figure 3A of [19]), even though CA3 is a monosynaptic target of DG [50]. However, for large morphs (environments 1 to 5 … or 7 to 3 …), the observed PV changes were the same for the two regions.

To simulate these morphing experiments, we constructed a model of the DG and CA3 networks (Figure 1A). CA3 cells were modeled as having input from DG, both lateral and medial parts of the EC, and recurrent excitatory input from other CA3 cells. DG cells were modeled as having input only from the EC. We modeled these inputs using a realistic number of contacts and realistic synaptic strength distributions (see Methods). Feedback inhibition was modeled separately in DG and CA3, giving rise to gamma frequency oscillations, as observed in these structures [29], [51], [52]. The delay of feedback inhibition (3.3±0.4 msec) [28] was made slower than that of recurrent excitation (1 msec) [25], [37]. Memories of environments 1 and 7 were set in the recurrent CA3 connections (see Methods). Rate was determined as follows (Figure 1B). The cell with strongest input will be the first to fire in a gamma cycle, triggering feedback inhibition. Other neurons that reach threshold may fire at some later time; still others with low excitation are unlikely to fire at all. However, because of noise in the system, firing is determined probabilistically. We thus take this probability of firing during a gamma cycle as measure of rate (see Methods).

### Simulation of the DG and CA3 model

With this biologically constrained model, we computed the activity of CA3 and DG cells in different morph states by analyzing the spike probability as the simulated rat traversed the environment (paths were taken from experimental data [38], [53]). The rat's location was represented by the activity of grid cells of the medial entorhinal cortex (MEC) (Figure 1C, left) [38], [53], whereas sensory information about the walls of the environment was represented by the activity of the cells of the lateral entorhinal cortex (LEC) (Figure 1C, right) [42], [43]. Both MEC and LEC maps were constrained by data (see Methods). Rate maps were computed from the simulated neural activity and the trajectories (Figure 1D). There were three open variables that we could not obtain from the literature: the relative strength of the input from LEC or MEC (α); the ratio of DG-to-EC input (β); and strength of the recurrent synapses (1-δ). We estimated these parameters computationally by searching the best fit to the experimental data using as reference the available metrics of both population and single-unit activity. This strategy allowed a direct comparison between the simulated data and experimental data using exactly the same methods. If the reader is not interested in the technical issue of parametrical optimization, he or she may wish to go directly to the next section, where we apply the model to the morphing data and analyze the evidence of attractor dynamics in the CA3.

**Figure 2:**
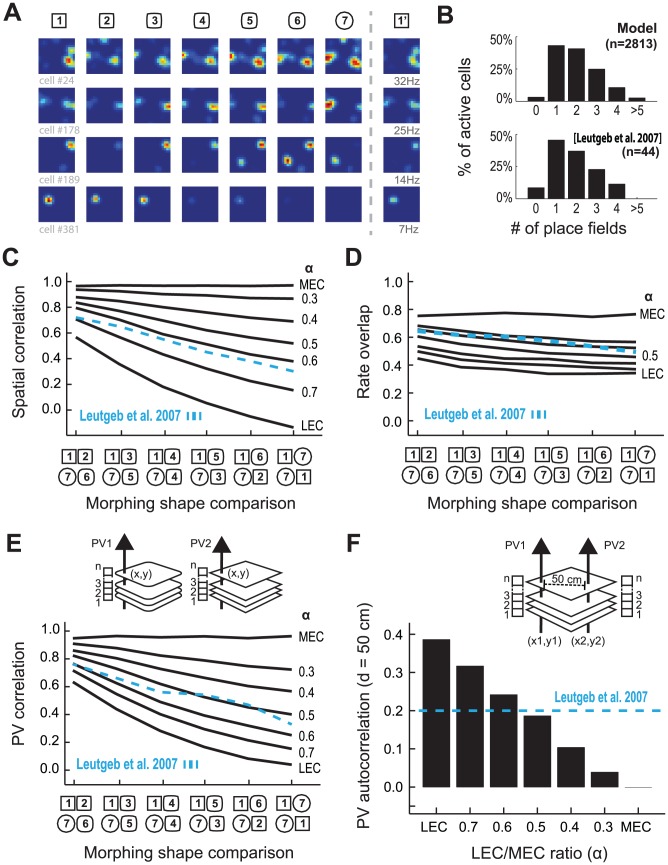
Rate remapping in simulated DG neurons reproduces the main features of experimental data. (**A**) Rate maps of selected simulated DG cells shown for different morphing shapes. Rate ranges from zero (blue) to maximum rate of specific cell in all shapes (red) (see Methods for the definition of the maximum rate). (**B**) Histogram of the number of place fields in each active DG cell in simulation (top) and in experimental data [19, bottom]. (**C**) Mean spatial correlation and (**D**) mean rate overlap between rate maps of the same cell in different environments; (**E**) PV correlation response to morphing, computed as the correlation between vectors of activity (PV1 and PV2) computed at the same position (x, y) but on different sessions; and (**F**) PV autocorrelation for bins 50 cm away, computed as the correlation between vectors of activity (PV1 and PV2) obtained at the different positions (x_1_, y_1_ and x_2_, y_2_) on the same sessions, as a function of α, the MEC/LEC ratio (solid black line), with overlaid experimentally observed values [19, dashed blue line].

Through the parametric optimization of the relative strength of the input from LEC or MEC (α), the simulated DG population data reproduced the main features of the experimental data (Figure 2). In our simulations, an average of 3.5% of DG cells were active at each session, in accordance with experimental measurements [47], [48]. DG cells exhibited place fields that independently rate remapped during morphing (Figure 2A), as observed in previous modeling studies [31], [34] and in the experimental data [19]. The distribution of the number of place fields per active cell was similar to that observed experimentally (Figure 2B). Simulated place fields had a peak rate of 11.92 Hz±7.87, comparable to 11.54 Hz±8.16 in Leutgeb et al. (2007).

The parameter α was optimized by searching in the range of valid α values (0–1) the value with which the simulated neural activity of the DG would better fit experimental data [19]. Both individual neurons, cumulative change in the average firing rate (rate overlap) and spatial correlation, and population activity metrics, PV correlation and PV autocorrelation, were used as metrics for the optimization process (see Methods). When we analyzed the activity of individual neurons, we observed that, for small and large morphs, strengthening LEC influence (high α) led to a higher decorrelation between firing rate distributions of individual cells than when MEC influence was high (low α) (Figure 2C). In the extreme case when considering only the LEC input (α = 1), the rate maps of individual DG cells in the two extreme shapes were uncorrelated. Strong LEC influence led to a higher change in the average firing rate of individual cells (lower rate overlap) compared to the condition of strong MEC influence (Figure 2D). If we interpret these results in terms of rate remapping (in which non-spatial information is encoded in the rate of spatially stable place fields) [34], [54], we observe that there is a trade-off between the ability of the DG cells to encode the wall shape information in the peak rate of place fields and the maintenance of the position of place fields. Interestingly, the experimental observation indicates that there is a compromise with balanced MEC and LEC influence (Figure S5 of [19]).

When we analyzed the population activity of DG neurons, we observed that stronger LEC contribution yielded stronger PV decorrelation for every pair of box shapes if compared to conditions in which the MEC input was greater (Figure 2E) with the best model fit, as in the analysis of single cells, obtained with a balanced MEC and LEC input (α = 0.5). We next investigated whether the encoding of the wall shape information disturbed the ability of the DG population to produce orthogonal representations of unrelated positions by measuring the autocorrelation of PVs obtained in the same box shape but at positions located 50 cm away (Figure 2F). High correlation would indicate a high overlap between representations of different positions, and a lower correlation would imply otherwise. We observed that strong LEC input resulted in PVs more strongly correlated at distant positions if compared to the condition with higher MEC influence. This observation indicates that also at the population level there is a trade-off between the ability of the DG to encode a specific position and a wall shape. Importantly, considering all population and individual cell metrics, an input with balanced MEC and LEC contribution provided the best fit to the experimental data [19].

**Figure 3:**
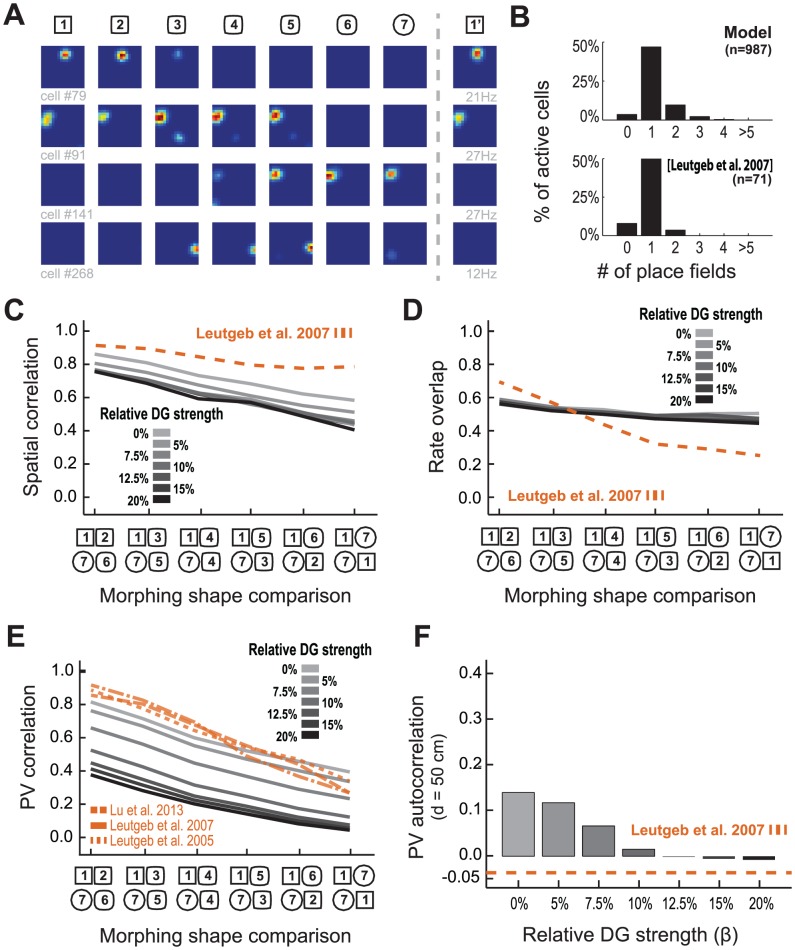
Rate remapping in simulated CA3 neurons without recurrent collaterals does not reproduce main features of experimental data. (**A**) Rate color maps of selected simulated CA3 cells shown for different morphing shapes. Rate ranges from zero (blue) to maximum rate of specific cell in all shapes (red). (**B**) Histogram of the number of place fields in each active CA3 cell in simulation (top) and in experimental data [19, bottom]. (**C**) Mean spatial correlation and (**D**) mean rate overlap between rate maps of the same cell in different environments; (**E**) PV correlation curve response to morphing; (**F**) PV autocorrelation for bins distanced 50 cm away in CA3 as a function of 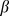, the DG input strength relative to EC input (solid black/gray line), with overlaid experimentally observed curve [19, dashed blue line].

Having established how to correctly simulate the DG and thus its input to CA3, we analyzed CA3 responses during morphing. We first analyzed the CA3 population response without recurrent connections. In our simulations of such a network, CA3 cells exhibited several properties consistent with the data. The distribution of the number of place fields per active cell were similar to that observed experimentally (Figure 3A,B). Peak place field firing was at 12.45 Hz±7.73 in simulation, which is comparable to 13.13 Hz±7.97 reported by Leutgeb et al. (2007). However, although rate remapping was observed during morphing, it was not consistent with the experimental data (Figure 3C–F), and this was true irrespective of the ratio (β) of DG-to-EC input. With increase of β, there was a general reduction of the correlation between rate maps in different environments (Figure 3C) and virtually no change in the average rate of the cells (Figure 3D). With respect to the population response to morphing, high values of β resulted in an overall increase of PV decorrelation when compared to the condition with low β (Figure 3E), thus not fitting the data [18]–[20]. Yet stronger DG input decreased the CA3 PV autocorrelation in distant positions (Figure 3F). In conclusion, we were unable to fit the CA3 data using a model without recurrent collaterals.

**Figure 4:**
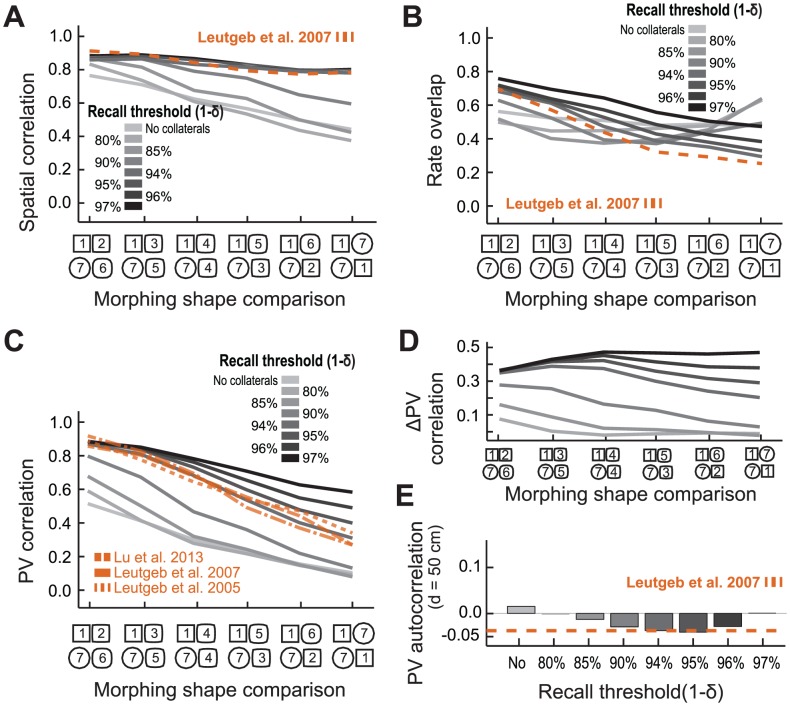
Rate remapping in simulated CA3 neurons with recurrent collaterals reproduces the main features of experimental data. (**A**) Mean spatial correlation and (**B**) mean rate overlap between rate maps of the same cell in different environments; (**C**) PV correlation curve response to morphing; (**D**) difference in PV correlation to the population response without recurrent collaterals; (**E**) PV autocorrelation for bins distanced 50 cm away in CA3 as a function of 1-δ, the recall threshold (solid black/gray line), with overlaid experimentally observed curve [19, dashed blue line].

We next analyzed whether the morphing data could be accounted for if recurrent collaterals were included (Figure 4). Synaptic weights of the CA3 recurrent collaterals were set based on the population activity in environments 1 and 7 (see Methods), emulating the experimental protocol in which the animals were familiarized to the two extreme shapes before the experiments. The addition of the excitatory feedback from the recurrent collaterals did not impair the formation of place fields and their ability to rate remap. Importantly, by increasing the strength of the recurrent synapses (1-δ), there was an increase of the correlation between rate maps of the same cell between different environments (throughout all morphs), leading to an almost flat response, as observed experimentally (Figure 4A). Such enhanced stability of the firing rate distribution of individual cells indicates that the place fields are present and unmoved throughout the morphing.

We next examined whether single cells were still responsive to morphing by measuring the cumulative change in the average firing rate of individual cells as morphing progressed. We found that the addition of the recurrent collaterals affected the average change in rate differently for small and large morphs (Figure 4B): for small morphs, the average change in rate was less than in the condition without recurrent collaterals; for large morphs, the average change in rate was higher than in the condition without recurrent collaterals. This indicates that not only are different wall shape conditions successfully encoded in the individual cells rate maps, but also that, for very similar inputs, the system attracts the average rate response to the stored pattern. Thus, the addition of the recurrent collaterals favors a code in which the information about the environment is encoded by the peak rate of place fields located at fixed positions. We found similar results when analyzing the population response to morphing: there was an overall increase in the PV correlation measured between sessions with different wall shapes approximating experimental observations (Figure 4C). For the parameters that led to the best model fit (1-δ = 95%), there was a stronger increase in the PV correlation for the small morph than for the large morph (Figure 4D). Moreover, we observed an additional reduction in the PV autocorrelation obtained in distant positions, approximating the observed value (Figure 4E). This indicates that the activity of the recurrent collaterals enhances the ability of the network to discriminate between unrelated positions. Altogether, these analyses show that the addition of recurrent collaterals allows an accurate description of rate remapping as seen in both the single-cell and population responses.

### Evidence of attractor dynamics in the comparison of DG and CA3 neural activity during morphing

**Figure 5:**
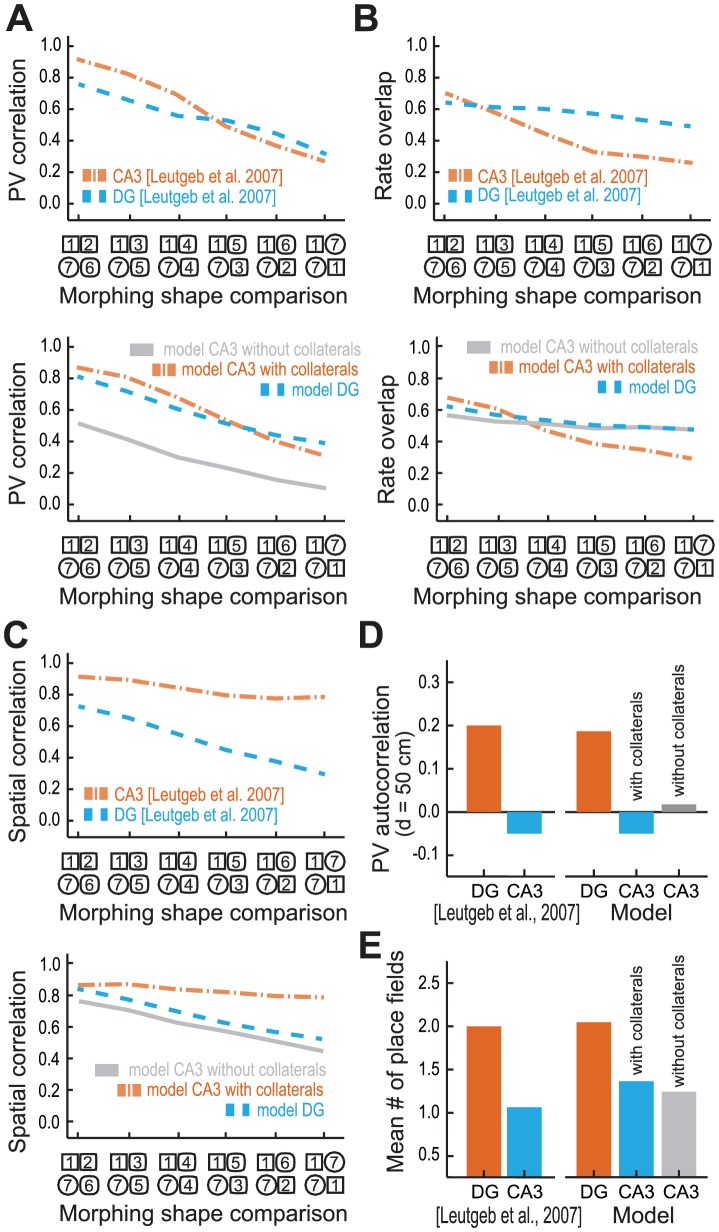
Recurrent collaterals explain the differences in the response of DG and CA3 to morphing observed experimentally [19]. (**A**) Progressive change in the PV correlation of DG (dashed blue line) and CA3 (dashed orange line). (**B**) Average rate overlap and (**C**) mean spatial correlation of individual cells rate maps as a function of morphing as observed experimentally (top) and its equivalent analysis of simulated data with best model fit (bottom), including the response of CA3 without recurrent collaterals (light gray line). (**D**) PV autocorrelation for large distances (50 cm) measured experimentally (left) and with model best fit (right). (**E**) Mean number of place fields measured experimentally (left) and with model best fit (right).

With all parameters set, we next directly compared the response to morphing in DG and CA3. The simulated data not only provided a model fit of the individual region response to morphing, but also provided a reasonable description of the relation between the population response of the CA3 and the DG to morphing (Figure 5). The experimental finding that DG population activity was more strongly affected by the small morph than the CA3 population activity was only observed when recurrent collaterals were present (Figure 5A). Notably, although the CA3 PV correlation was increased during both small and large morphs by the recurrent collaterals, this effect was stronger for small morphs than for large morphs, indicating the existence of a basin of attraction (ΔPV correlation of 0.35 for small morph against 0.20 for large morph, Figure 4D). Further, we found additional evidence for a basin of attraction by analyzing how single CA3 cells changed their firing rate throughout morphing (Figure 5B); for the large morphs (1–7) in which little effect of the attractor dynamics is expected, we found a higher change in the average firing rate in CA3 when compared to DG in the presence of recurrent collaterals (rate overlap in CA3 is ∼0.2 lower than in DG), setting the baseline of how the rate of DG and CA3 cells is affected by morphing. For the small morphs (1–2), the condition in which the attractor dynamics would be effective, in the CA3 there was a lower change (rate overlap in CA3 is ∼0.05 higher than in DG) in the average firing rate in CA3 than in DG when recurrent collaterals were included. These results indicate that, even though CA3 cells are naturally more sensitive to changes in the environment when it is out of a basin of attraction (as seen by the baseline results of the large morph), when we consider the conditions in which attractor dynamics are effective, there is a lower sensitivity to change in the CA3 cells. Notably, we also found that the addition of collaterals contributed to the spatial stability of place fields in the CA3; only in the presence of recurrent collaterals were individual rate maps of CA3 cells less affected by morphing than individual rate maps of DG cells (Figure 5C). The analysis of the dynamics of CA3 rate coding also revealed the role of the feedforward excitation and competitive inhibition in pattern separation, as there is a considerable reduction in the PV autocorrelation between two distant and unrelated areas (Figure 5D). Also, consistent with previous findings that a two-stage process increases spatial specificity [35], we observe a reduction of the mean number of place fields in CA3 (Figure 5E). These results allow the identification of the specific role of the neural circuits of DG and CA3 in memory: while the convergence of excitatory feedforward input and the internal inhibitory competition cause pattern separation, the recurrent excitation has a major role in pattern completion.

Importantly, neither in the reported data nor in the simulation was there any evidence that morphing produced a sharp rather than a graded transition in any of the computed measurements (Figure 5A). Thus, three important conclusions follow. First, the recurrent connections in CA3 do have an attractor function; during small morphs, they “attract” the dynamics toward a stored pattern (e.g., the square or the round shape). Second, this attractor dynamics modulates rate remapping, thereby leaving the spatial information intact. Third, despite this attractor function, the CA3 firing pattern undergoes a graded rather than abrupt change during morphing over intermediate states (i.e., 4 and 5).

**Figure 6:**
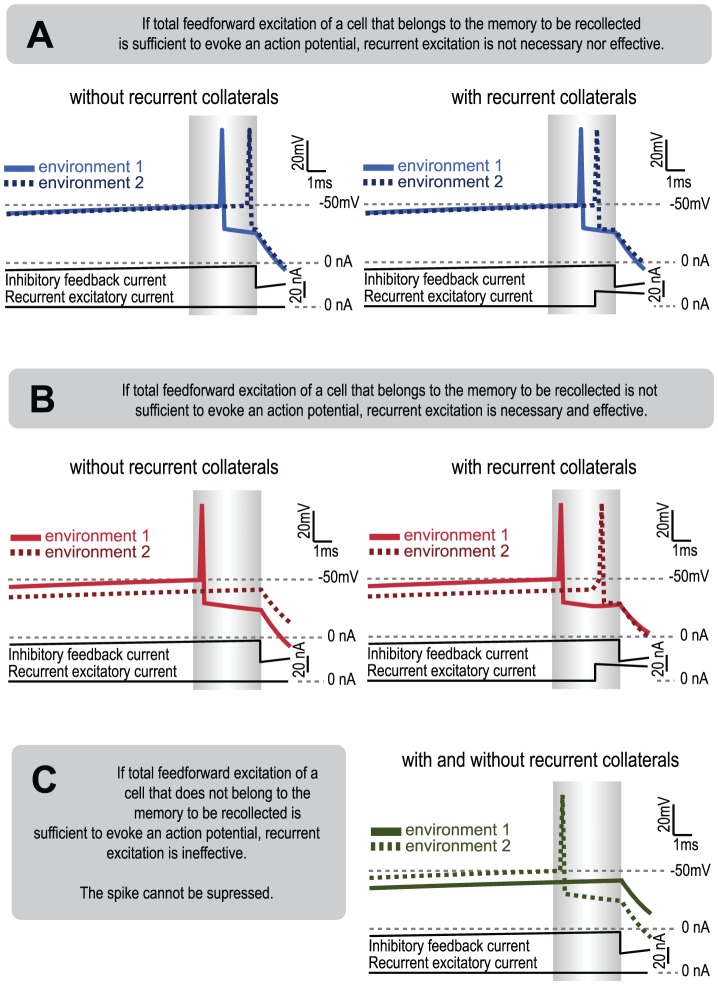
Dynamics of single cells during a small morph in the presence and absence of recurrent excitation. (**A**) Trace of a single gamma cycle of a representative cell of the CA3 memory in environments 1 (solid line) and 2 (dotted line). For both wall shapes, an action potential is released both in the absence of active recurrent collaterals (left plot) and in their presence (right plot). Time is represented by the horizontal axis. Gray area designates the window between the first spike in the population and the onset of global inhibition. Cell voltage and input currents are shown on the ordinate. (**B**) Trace of a representative cell of the activated CA3 memory in environment 1 (solid line) and environment 2 (dotted line). Pattern completion can be observed in the presence of recurrent collaterals (right plot), but not in their absence (left plot). (**C**) Trace of a representative cell not included in the memory (solid line) and with high excitation in the small morph (dotted line). Recurrent collaterals are not effective because the cell is not part of an active memory. There is no mechanism to avoid any action potential released by cells not included in a pattern.

To characterize the mechanisms by which the recurrent collaterals affect the population response to morphing, we analyzed the dynamics of single cells during small morphs in the presence and absence of recurrent excitation. The small morph is a condition in which there should be a moderate but still noticeable change in the input pattern to CA3. In the presence of attractor dynamics, the input pattern will be within the stored pattern basin of attraction and thus pattern completion should be observed. We analyzed the firing pattern produced under these conditions and the subsequent influence of recurrent excitation. The small morph had three important effects (Figure 6). First, in cells whose total feedforward input, including the excitatory current from EC and DG, was strong (0.9 nA) and led to a spike, the presence of recurrent excitation did not yield a significant increase in the probability of an action potential (Figure 6A). Second, in CA3 cells that were part of the stored pattern but received DG/EC input after morphing that was subthreshold (0.6 nA), the recurrent input triggered a spike, thereby producing pattern completion (Figure 6B). In the absence of recurrent excitation, such cells would not fire. This explains why there is a higher PV correlation between the population responses to a stored pattern and a small morph in the presence rather than in the absence of recurrent excitation (Figure 5A). Thus, in this way, the internal dynamics provided by the CA3 recurrent synapses attracts a cell toward a stored pattern, thereby producing rapid pattern completion within a single gamma cycle [25]. Third, what the dynamics of the attractor cannot do is erase spikes that have already occurred. Consider that, after a small morph, a cell is strongly excited by DG/EC that is not part of the nearby stored pattern (Figure 6C). Because this spike has occurred and cannot be erased, the total activity during the short firing period cannot be identical to the stored pattern. Likewise, activity induced by additive noise cannot be suppressed. Thus, although recurrent excitation can attract CA3 to a stored pattern, this attraction cannot be perfect.

### Evidence of experience-dependent plasticity in the CA3 collaterals

**Figure 7:**
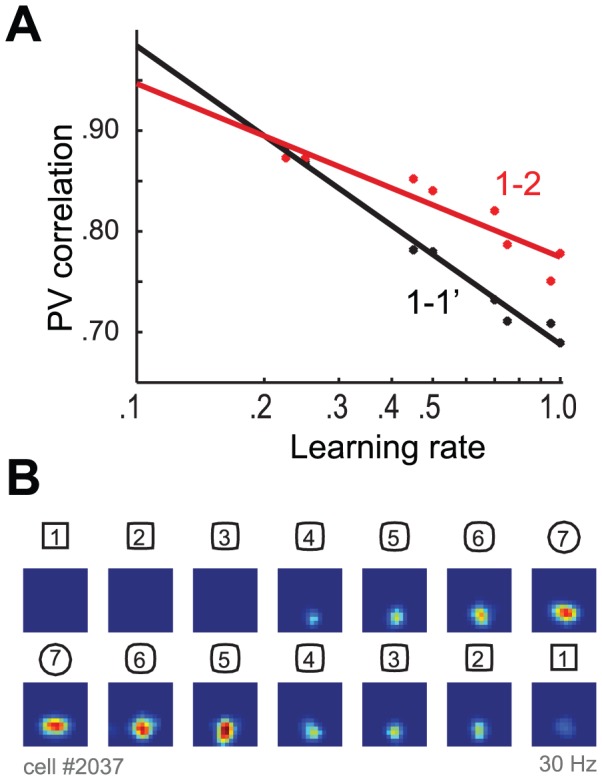
The number of intermediate sessions affects the quality of pattern reconstruction. (**A**) PV correlation between smallest morph (1-2) and two successive recordings of the first environment (1-1′) as a function of the learning rate. For 1-2, there is no intermediate session, whereas for the 1-1′, there are five intermediate sessions (2–5). With a learning rate of about .03, the measured PV correlation is the same for 1-1′ and 1-2 within one full session. (**B**) Rate maps of the simulated activity of a sample CA3 cell during regular and inverse morphing showing hysteresis.

The understanding that attractor dynamics cannot eliminate spikes that are not part of the stored pattern has further implications. In the morphing experiments, the smallest morphs (1, 2) displayed a PV less than 1 (0.9). However, because two measurements in environment 1 (albeit with intermediate sessions in all other environments, i.e., 1-1′ is obtained from the sequence 1-2-3-4-5-6-7-1 with six intermediate sessions) also showed a PV correlation of 0.9 [19], it was suggested that an attractor mechanism made the response in environment 2 identical to that in environment 1. Our analysis, however, suggests that such perfect attractor reconstruction cannot occur, and we suggest an alternative explanation: that the intermediate sessions between the two recordings in environment 1 altered the stored attractor, thereby reducing the correlation in the 1-1′ morphing to 0.9. Thus, the 1-2 environments evoked different responses because the attractor system does not work perfectly as explained above, whereas the 1-1′ environments evoked different responses because of the learning produced in intermediate environments. We simulated the morphing procedure with varying learning rates and observed that the 1-1′ (1-2-3-4-5-6-7-1) and the 1-2 correlation were not equally affected by the exposure to different environments (Figure 7A). Interestingly, because of the sequence in which the wall shapes were changed, the correlation of the 1-1 morphing changed more thoroughly to higher learning rates due to the fact that there were more intermediate trials (n = 6) between the comparisons when compared to the correlation of the 1-2 environments (n = 0), which allowed that, for a specific learning rate, both comparisons are equivalent.

Subsequent work supports our interpretation: when 1-1 comparisons are made without exposure to intermediate environments, the PV correlation was higher (0.93 in Figure 5 of [20], 0.96 in Figure S5 of [55]). In the same studies, the PV correlation was lower [0.90 in 20,0.91 in 55] if there were intermediate exposures to other environments and was progressively reduced with the number of such exposures, as would be predicted if these exposures produced learning and a modification of the stored attractors. Further evidence of experience-dependent plasticity in the recurrent collaterals is that hysteresis was observed in CA3, but not in the DG [19]. In our simulations with learning in the recurrent collaterals, we observed comparable levels of hysteresis in the CA3 rate maps (Figure 7B).

### Dependence of place cells remapping on grid cell stability

**Figure 8:**
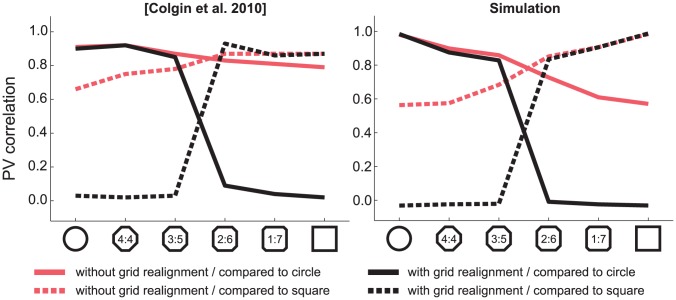
Effect of grid cell realignment on the CA3 population response to morphing. Reproduction of the results of Colgin et al. [21] (left box) is compared to our experimental results (right box). In the condition in which the animals are trained in the same arena at the same location (red line), morphing induces a graded decrease in population correlation if compared to the initial state (red solid line) and a graded increase in correlation if compared to the final state (red dotted line). Under this condition, grid cells do not realign [19], [40]. In the condition in which the animals are trained in different arenas or in the same arena but at different locations (black line), morphing induces a sharp and deep decrease in correlation if compared to the initial state (black solid line) and a graded increase in correlation if compared to the final state (black dotted line). Under this condition, grid cells realign [40].

We next investigated how place cells respond under conditions in which environmental change does produce grid cell realignment. We investigated how grid cell realignment affects the population response to morphing in the CA3. Grid cells were shown to realign when the animal is trained at the same location but in different boxes or at different locations but with the same box [40]. Under these conditions, place fields do not remain stable at the same positions, characterizing global remapping [40], [54]. During morphing, grid cells seems to realign at an intermediate position, causing an abrupt change in the CA3 population neuronal activity [21], [56] (Figure 8, left). To verify whether our model produces results in accordance to the literature, we realigned the grid cell population in the middle of morphing (see Methods) and computed the activity of CA3 cells (Figure 8, right). We observed that, following the realignment of the grid cells, there was an intense and abrupt change in the PV correlation. This effect is further supported by the observation that the change in the PV correlation during morphing is graded when grid cells are stable [19]. Our data thus corroborate the view that grid cell stability is required for rate remapping in the DG and CA3.

## Discussion

We have addressed the question of whether the CA3 memory system can be considered an attractor network in the face of ostensibly conflicting experimental results. Using a simulation of the EC/DG/CA3 system, we show that firing patterns recorded in CA3 during the morphing of an environment are in accord with what is expected if CA3 is an attractor network. When the environment is subject to small morphs, DG granule cells, which do not have recurrent synapses, change their firing patterns substantially. In contrast, CA3 cells, which do share recurrent plastic connections, change much less, indicating an attraction to a stored pattern. Importantly, our simulated observations are in accord with experimental data [19], [57]. Given that DG provides strong input to CA3 [50], attraction of CA3 cells to a stored pattern must be due to recurrent activity within CA3 itself. Our simulations show that the recurrent collaterals of CA3 can produce these dynamics and do so within a short time interval consistent with the theta-phase specific firing of CA3 cells [24].

Importantly, our work clarifies the issue of whether sharp transitions during morphing are a requirement for demonstrating that a network follows an attractor dynamic. The argument that CA3 might not be an attractor network [21] was based on the observation that sharp transitions in PV correlation did not occur during morphing, thus not displaying a criterion of attractor networks. This criterion was suggested by work in which attractor networks were activated by brief external inputs and were then allowed to evolve to a stored pattern after the external input was removed [2], [11], [12]. The sharp transition occurs because without external input, attractor networks are all-or-none; with dynamics unconstrained by external input, the network uses internal dynamics to converge to the closest of the stored memories. For this reason, the final state of the network does not show intermediate states, and sharp transitions are expected. Such a feature is, however, not applicable to the hippocampus because external input from the EC and from DG to CA3 is never absent. Under these conditions, our simulations show that sharp transitions do not occur (Figure 5C and 6A). Thus, under the conditions of the morphing experiment, sharp transitions are not an appropriate criterion for identifying an attractor network.

There is a specific case in which sharp transition can be observed in the CA3 [21], [56]: if the animal is familiarized with the two extreme shapes with altered distal cues, a different spatial coordinate system is assigned for each memory. As the EC globally remaps with different distal cues, a sharp transition in the CA3 will occur but will be caused by changes in cortical activity and cannot be attributed to attractor dynamics in CA3 [40]. In the experiments that we have analyzed here, distal cues were kept unaltered, and this prevents global remapping in MEC.

For CA3 to function as an associative memory, the recurrent synapses must be able to undergo activity-dependent changes in their synaptic strength. Indeed, work in the slice preparation has clearly shown that these synapses can undergo long-term potentiation (LTP) [9], [10], but there has been no previous *in vivo* demonstration that these synapses can change in response to environmental stimuli. We argue that aspects of the data reported by Leutgeb et al. [19] strongly argue that the attractors formed in CA3 are continuously subject to learning. Indeed, this is demonstrated by the fact that exposing rats to intermediate environments is sufficient to produce a modest change in CA3 PV correlation and thus its synapses (Figure 7) [20], [55]. The key observation is hysteresis of the PV; if an altered environment is interposed between two test sessions in the same environment, the PV in the two identical environments will be slightly altered. Importantly, this hysteresis is not observed in DG [19], strongly suggesting that it occurs because of the plasticity within the recurrent connections of CA3. Indeed, we are able to reproduce these hysteresis effects in our model that simulates the effects of experience-dependent Hebbian plasticity in the CA3 excitatory recurrent connections.

This analysis of morphing suggests future experiments investigating the role of attractors and their modification by learning. Given that attractor dynamics can now be more precisely identified, it would be of interest to test directly whether NMDAR action during learning of the square/round environments is necessary for attractor formation, as would be predicted based on *in vitro* studies analyzing pattern completion [58]. Indeed, following this prediction, NMDAR seems to be required during memory formation, as shown by the fact that pattern completion during subsequent recall is prevented [59]. NMDARs are not required during memory recall [60]. This is consistent with the observation that the latter effect depends on the fast dynamics of our model. Additionally, a second type of analysis could investigate discretization during learning [61]; it has previously not been possible to experimentally address the question of how finely the world is divided, but it is now approachable through the study of CA3 attractors in particular, by addressing both the temporal and the spatial ranges of this memory segmentation. In addition, we can speculate that GABA-dependent dendritic shunting of spike-time-dependent learning can assure that also the learning dynamics is restricted to single gamma cycles [62]. From the model presented here, we will be able to estimate the average size of the population of CA3 neurons that define a distinct memory, their interrelation, and the drift that they might be subject to. In addition, their embedding in a theta-gamma code raises the question of whether single memory segments defined in a single gamma cycle are, in turn, integrated in hierarchical structures following the theta rhythm. Further, the drift of CA3 memory that we have identified would suggest that, for a more permanent storage of memory segments, other structures will have to be engaged to solve the so-called plasticity-stability dilemma [63]. Finally, the ability of rather short exposures to altered environments to change the attractor properties of CA3 facilitates the study of learning in a defined network. This may allow the analysis of the spike patterns that lead to learning, the role of neuromodulators, and the role of repetition/replay in producing long-lasting synaptic modification.

The demonstration that CA3 cells display the properties expected of an attractor network carries special significance because it provides the key remaining evidence, i.e., analysis of *in vivo* data, that is necessary to establish the associative memory function of CA3. As discussed, the existence of modifiable recurrent connections in CA3 suggested that CA3 is an attractor network. Consistent with this hypothesis, a mutation that disables synaptic plasticity in CA3 prevents behavior that is dependent on pattern completion [59], [64]. Additionally, signatures of experience-dependent plasticity and pattern completion have been obtained *in vitro* with CA3 slices [58]. Thus, taken together, the anatomy, the behavioral experiments, *in vitro* electrophysiology, and our analysis of *in vivo* recordings make a strong case that CA3 is, in fact, an associative memory structure that follows attractor dynamics. The CA3 network analyzed here is thus among the very few cases in which the evidence regarding network, cellular, and anatomical properties has converged to explain an important aspect of memory and behavior.
